# Taphonomic Analysis of the Faunal Assemblage Associated with the Hominins (*Australopithecus sediba*) from the Early Pleistocene Cave Deposits of Malapa, South Africa

**DOI:** 10.1371/journal.pone.0126904

**Published:** 2015-06-10

**Authors:** Aurore Val, Paul H. G. M. Dirks, Lucinda R. Backwell, Francesco d’Errico, Lee R. Berger

**Affiliations:** 1 Evolutionary Studies Institute, University of the Witwatersrand, Johannesburg, South Africa; 2 Department of Science and Technology/National Research Foundation Centre of Excellence in Palaeosciences, University of the Witwatersrand, Johannesburg, South Africa; 3 School of Earth and Environmental Sciences, James Cook University, Townsville, Australia; 4 School of Geosciences, University of the Witwatersrand, Johannesburg, South Africa; 5 Centre National de la Recherche Scientifique, Unité Mixte de Recherche 5199 PACEA, Université de Bordeaux, Talence, France; 6 Department of Archaeology, History, Cultural Studies and Religion, University of Bergen, Bergen, Norway; University of Oxford, UNITED KINGDOM

## Abstract

Here we present the results of a taphonomic study of the faunal assemblage associated with the hominin fossils (*Australopithecus sediba*) from the Malapa site. Results include estimation of body part representation, mortality profiles, type of fragmentation, identification of breakage patterns, and microscopic analysis of bone surfaces. The diversity of the faunal spectrum, presence of animals with climbing proclivities, abundance of complete and/or articulated specimens, occurrence of antimeric sets of elements, and lack of carnivore-modified bones, indicate that animals accumulated via a natural death trap leading to an area of the cave system with no access to mammalian scavengers. The co-occurrence of well preserved fossils, carnivore coprolites, deciduous teeth of brown hyaena, and some highly fragmented and poorly preserved remains supports the hypothesis of a mixing of sediments coming from distinct chambers, which collected at the bottom of the cave system through the action of periodic water flow. This combination of taphonomic features explains the remarkable state of preservation of the hominin fossils as well as some of the associated faunal material.

## Introduction

The dolomitic caves in the Cradle of Humankind, South Africa, have yielded extremely rich late Pliocene to early Pleistocene palaeontological assemblages, which comprise fossils of various hominin taxa (*Australopithecus africanus*, *Australopithecus prometheus*, *Paranthropus robustus*, early *Homo* and *Homo ergaster*) and associated mammals, reptiles, and birds (see for instance [1–13]). These faunal assemblages are usually composed of isolated and fragmentary skeletal remains, and include few, if any, hominin fossils, with the notable exceptions of Sterkfontein and Swartkrans [1–3, 6, 7, 13–15]. Modes of bone accumulation into the cave systems [1, 8, 16–20] lead to a generally poor state of preservation of the fossils. Hence, accumulation by carnivores, including extinct and extant felids and hyaenids, and birds of prey, as well as post-depositional damage induced by various biotic (e.g. porcupines) and abiotic agents (e.g. calcification/decalcification cycles, sub-aerial weathering, sediment reworking and sediment pressure) contribute to a high degree of fragmentation of skeletal elements, damaged bone surfaces, compaction and deformation of the bones, and absence of articulated remains.

**Fig 1:**
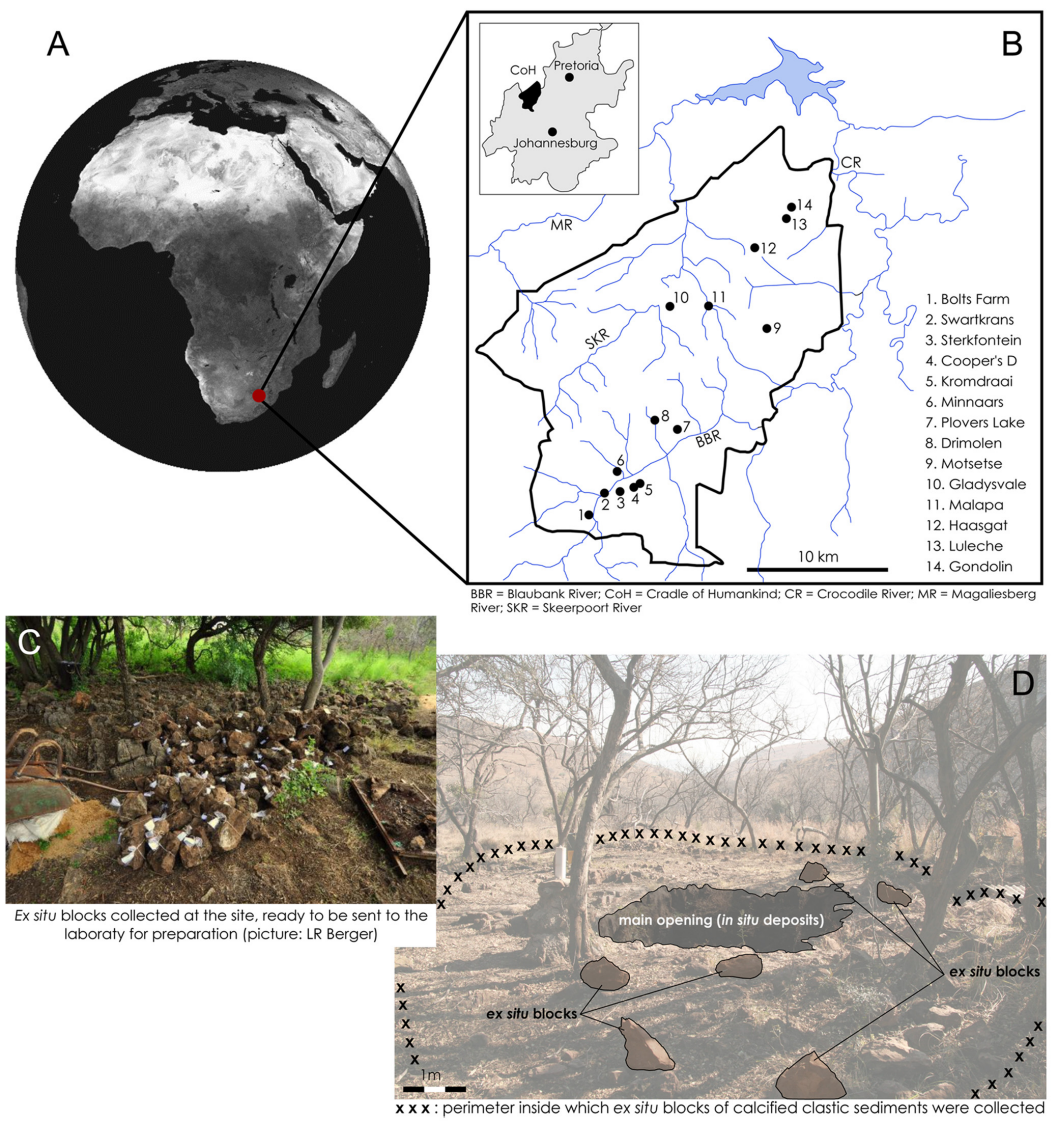
General presentation of Malapa. Top: location of Malapa in South Africa (A) and in the Cradle of Humankind (B). Bottom: pictures taken at the site during collect of *ex situ* blocks (C) and preliminary excavations of the *in situ* deposits (D), between 2008 and 2009.

In the context of the Plio-Pleistocene faunal assemblages from the Cradle of Humankind, the recently discovered deposits at Malapa (Fig 1) constitute a unique case. Not only have they yielded a very high number of hominin specimens (belonging to the recently named new species, *Australopithecus sediba*), the material is also extremely well preserved [21, 23]. This indicates a suite of taphonomic events different from those observed in other South African cave sites. As a preliminary hypothesis [22] it was proposed that the hominins entered the cave via a vertical shaft leading to a cave chamber offering no access to scavengers. The combination of a natural death trap scenario followed by a debris flow was suggested to explain the remarkable state of preservation of the hominins [22]. This hypothesis is now supported by a comparative taphonomic analysis of hominin and non-hominin remains provided here.

## Background to the Site

The Malapa fossil site is located 15 km northeast of the Sterkfontein Caves, within the Cradle of Humankind (World Heritage Site), at 25°53’42’S, 27°48’05’E. Faunal remains studied here, found associated with two *Au*. *sediba* skeletons (MH1, Malapa Hominin 1, a juvenile male; and MH2, Malapa Hominin 2, an adult female), were excavated from a pit, 3.3 m by 4.4. m in size and 4 m deep, which represents the erosional remnants of what was once a deep cave system [23]. Malapa was mined for flowstone in the early 20^th^ century [20, 24], which resulted in the removal of fossil-bearing blocks from the pit. These blocks were scattered around the pit and collected between 2008 and 2009 (Fig 1). Most of the fossils (n.1154) were manually extracted from *ex situ* blocks of clastic calcified sediment. Some specimens (n.148) were recovered *in situ* during preliminary excavations of the decalcified sediments in Facies D, E and F. The estimated volume of sediment from which *in situ* or *ex situ* fossils were recovered is small (<21.2 cubic meters). Fossils from other Plio-Pleistocene deposits from the Bloubank Valley in the Cradle of Humankind are usually recovered from ‘breccias’ or clastic calcified sediments. In this region, bones and carcasses have generally accumulated in the form of talus cones inside cavities in the dolomites. These talus cones calcified due to lime-bearing solutions dripping from the roof, which resulted in consolidated fossil-rich ‘breccias’ or clastic calcified sediments [1]. At Malapa, erosion of the roof of the cave led to the exposure of such sediments, which locally underwent decalcification due to soil forming processes involving the circulation of slightly acidic water.

Sedimentary evidence from Facies D, the deposit dated to 1.977 ± 0.002 Ma [25] that has yielded the bones of MH1 and MH2 [20, 21], indicates that it originated as a debris flow, whereas the overlying sediments belonging to Facies E and F were deposited as muddy accumulations along the cave floor, with the occasional influx of sandy material as a result of directional water flow [22, 25]. During much of their depositional history, the sediments in Facies E and F were water-logged. In terms of composition, the sediments in Facies D, E and F, represent a mixture of poorly-sorted, autochthonous (from within the cave), and allochthonous (external to the cave) clastic deposits including interspersed mudstone and muddy sandstone units that contain a high proportion of manganese-rich peloidal material, derived from the reworking of muddy sediment by insects (beetles, termites). These sediments display a general fining upward sequence, from coarser sand with breccia blocks at the base of Facies E, to pure mud at the top of Facies F, with isolated dolomite blocks and fossils embedded in this muddy facies [22, 25].

## Methods

The vertebrate assemblage was analyzed to determine the composition of the faunal spectrum, survival of skeletal elements, mortality profiles, and agents responsible for bone breakage and surface modifications. The NISP represents the total Number of Identified Specimens [26], with “specimen” referring to any bone or tooth fragment identified to the anatomical level [27] and/or the taxonomic level [28, 29]. In most cases, bones attributed to a family or a species have also been assigned to a specific skeletal element since taxonomic identification cannot be conducted without anatomical identification [30]. The MNE (Minimum Number of Elements) [31] is used to estimate the frequency of each skeletal element [30]. In our estimation of the MNE, the method chosen follows a manual overlap method as advocated by some [32], which takes into account criteria such as size and morphology. The criterion of age (infant, juvenile, adult, old) is also considered. The MNI, or “Minimum Number of Individuals necessary to account for all the kinds of skeletal elements found in the skeleton of a taxon” [30], is calculated in order to estimate the abundance of different taxa within the assemblage [33]. The MNI is estimated using the highest MNE value for each taxon and combines different criteria, such as age, size and morphology. The percentage survival is used to calculate the degree of bone preservation in the faunal assemblage and to obtain information about body part frequencies. We refer to Brain’s definition: the percentage survival is the “observed proportion of each anatomical part that survived attritional processes” [34, 35, 36] and is calculated as follows: (100 x MNE_e_) / (MNI x number of times ‘_e_’ occurs in one skeleton, where ‘_e_’ represents a given skeletal element).

For the description of breakage patterns, we use the criteria proposed by Villa and Mahieu [37] for human long bones to differentiate between green and dry bone breakage patterns. Since these criteria have been established on long bones, no breakage pattern is attributed to any other bone category. The fracture angle, outline and edge are considered, as well as the intensity of the fragmentation (i.e. shaft circumference, shaft fragmentation, lengths of the shaft fragments and breadth/length ratio). Fractures on dry bones are typically characterised by a right angle, a transverse outline and a jagged edge, whereas green bone fractures are associated with an oblique angle, curved outline and smooth edge [37].

Two levels of articulation for the Malapa fossils were defined. A “true articulation” refers to bones that are still directly associated with one another (direct contact, with no sediment between the bones), in their original anatomical position. The term “anatomical proximity” refers to bones that are preserved close to one another in the calcified sediment, but are no longer fully articulated, and have some sedimentary infiltrate between them. Microscopic analysis of the faunal assemblage was conducted for the identification and description of bone surface modifications, using an Olympus SZX 16 Multifocus microscope at magnifications between 7 and 115 times.

We thank the South African Heritage Resource agency for the research permits; the Nash family and John Nash Nature reserve for granting access and continued support of research at Malapa.

## Material

**Fig 2:**
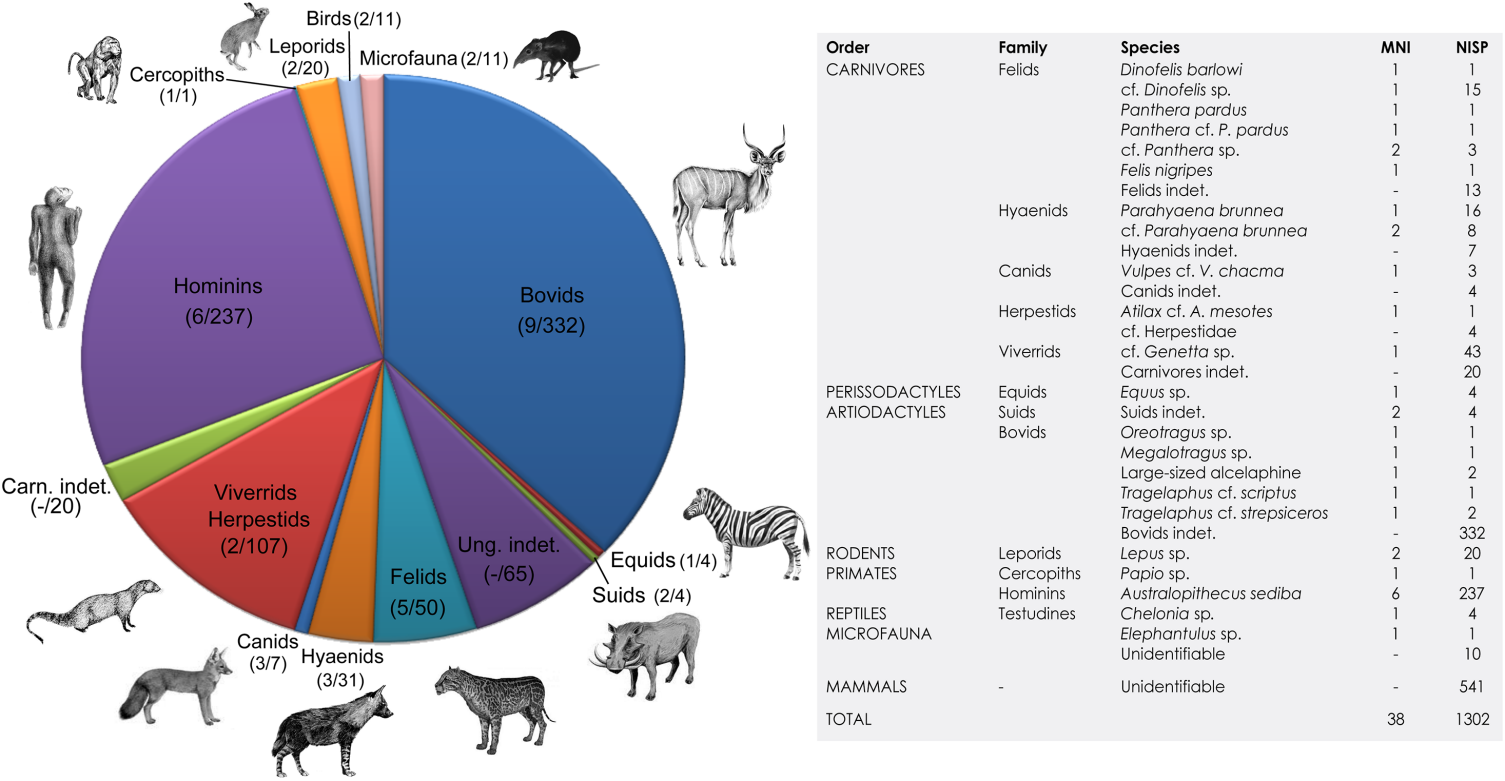
Composition of the fauna from Malapa. Values in brackets represent the minimum number of individuals.

The faunal assemblage to date, including hominin remains, comprises 1302 fossil specimens (bones, bone fragments, teeth, tooth fragments, horn cores, and carapace fragments; S1 Dataset). Abundant faunal remains are likely to be present inside *in situ* deposits that have still to be excavated, as well as in *ex situ* blocks awaiting preparation. It is therefore important to bear in mind that the faunal remains analyzed here represent a small sample of a much larger faunal assemblage at the site. Of the 1302 remains studied, 971 have been identified to family level, while 331 have been classified as unidentifiable mammal remains. The assemblage comprises 18 species of ungulates, carnivores, primates, rodents and birds (Fig 2) [22, 38, 39, 40]. In terms of body size, taxa range from microvertebrates (*Elephantulus* sp.) [39] to large ungulates (class size V) [22]. Non-hominin primates are represented only by a partial skull assigned to a cercopithecoid (*Papio angusticeps*) [40]. Carnivores (MNI: 14, MNE: 171, and NISP: 173), ungulates (MNI: 13, MNE: 306, and NISP: 434) and hominins (MNI: 6; MNE: 160, and NISP: 242) dominate the assemblage, with bovids representing the highest of number of identified specimens (Fig 2).

## Results

The Malapa fossil assemblage is characterized by a relatively high faunal diversity, with a total of 18 taxa identified. Animals with good climbing proclivities, namely carnivores and primates (mostly hominins) are well represented (Fig 2). Mortality profiles for the hominins, ungulates and carnivores (S5 Fig) closely resemble mass mortality patterns, normally associated with non-selective, catastrophic events [41].

**Fig 3:**
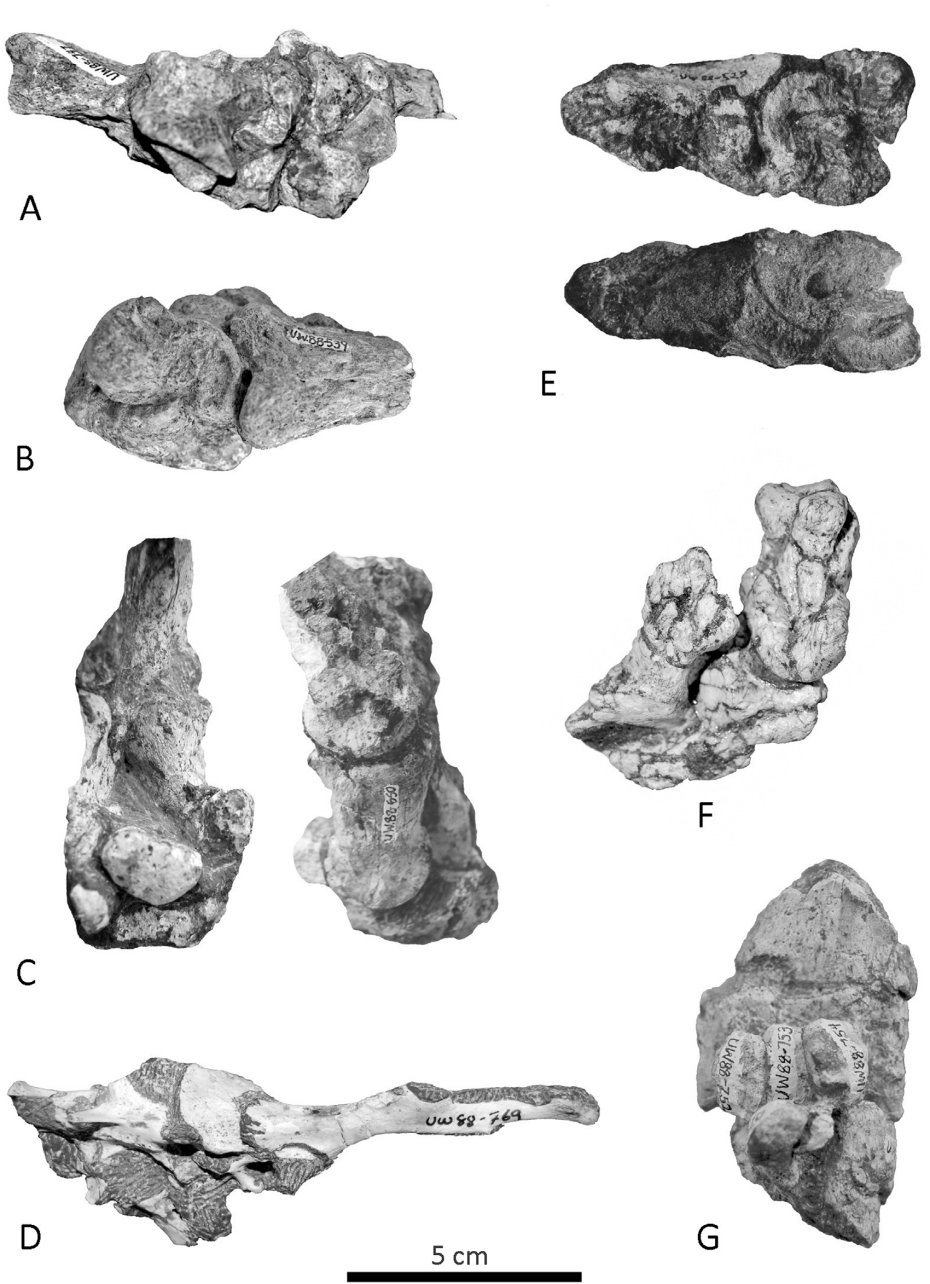
Examples of articulated faunal remains. A) *Dinofelis* sp. articulated right ankle (UW88-747). B) *Parahyaena brunnea* articulated ankle (UW88-739). C) Bovid articulated foot (UW88-650). D) *Lepus* sp. pelvis articulated with the sacrum and last lumbar vertebrae (UW88-769). E) Bovid articulated intermediate and distal phalanges and one sesamoid (UW88-528). F) Bovid intermediate and distal phalanges (no specimen number). G) Bovid metatarsal, first phalanx and sesamoids specimens (UW88-751-756).

The majority of the faunal remains show a good state of preservation. Body part representations for ungulates and primates do not reflect any particular selection pattern (no significant over-representation of specific skeletal elements, or under-representation or absence of others) and are therefore inconsistent with an assemblage accumulated by carnivores [42, 43] (S1 Fig). Instead, cranial and post-cranial elements are represented in the assemblage, including teeth, elements of the axial skeleton, limbs and extremities (phalanges, tarsals, carpals and caudal vertebrae) (S1 Dataset; S1 Fig; S1 Table). Skeletal profiles generally follow a density-mediated pattern [44–47], whereby denser and more compact bones (e.g. long bone shafts, tarsals, mandibles and metapodials) show percentages of survival greater than those of lighter and more fragile elements (e.g. ribs, vertebrae and scapula blades) (S1 Fig; S1 Table). Articulated and near-articulated remains of bovids, large and small carnivores, hominins and rodents have been recovered in abundance, and all types of joints, including persistent, intermediate and unstable joints (ankles, hands, feet, partial vertebral columns, knee and limbs) are represented (S2–S3 Tables; S2–S4 Figs; Fig 3). Apart from the skeletons of MH1 and MH2, 12 antimeric sets of bones belonging to small and large carnivores, bovids and leporids have been identified (S4 Table).

Despite the significant number of carnivore remains in the assemblage, and the presence of extant taxa known to accumulate bones inside caves (the leopard *Panthera pardus* [1, 48] and brown hyaena *Parahyaena brunnea* [49], as well as extinct false saber-tooth cat, *Dinofelis barlowi*, considered a potential bone accumulating agent [1, 50]), microscopic analysis of the bone surfaces revealed no carnivore tooth or gastric acid damage. Bone breakage patterns are consistent with fracture due to a fall, or naturally occurring on dry bones, rather than breakage by carnivores.

Similarly, porcupines are represented by two quills in the faunal assemblage, but marks produced by their teeth are absent from the faunal collection so far. There is no indication of hominin accumulation or modification of bone, and no evidence of damage by birds of prey. The principal biotic agents responsible for bone surface modification are invertebrates and microbes, detailed descriptions of which are in progress. The majority of the assemblage (65%) is only slightly weathered and falls within stages 1 and 2, *sensu* Behrensmeyer [51]: remains characterized by superficial or deeper cracking; cortical surface of the bones still preserved.

**Fig 4:**
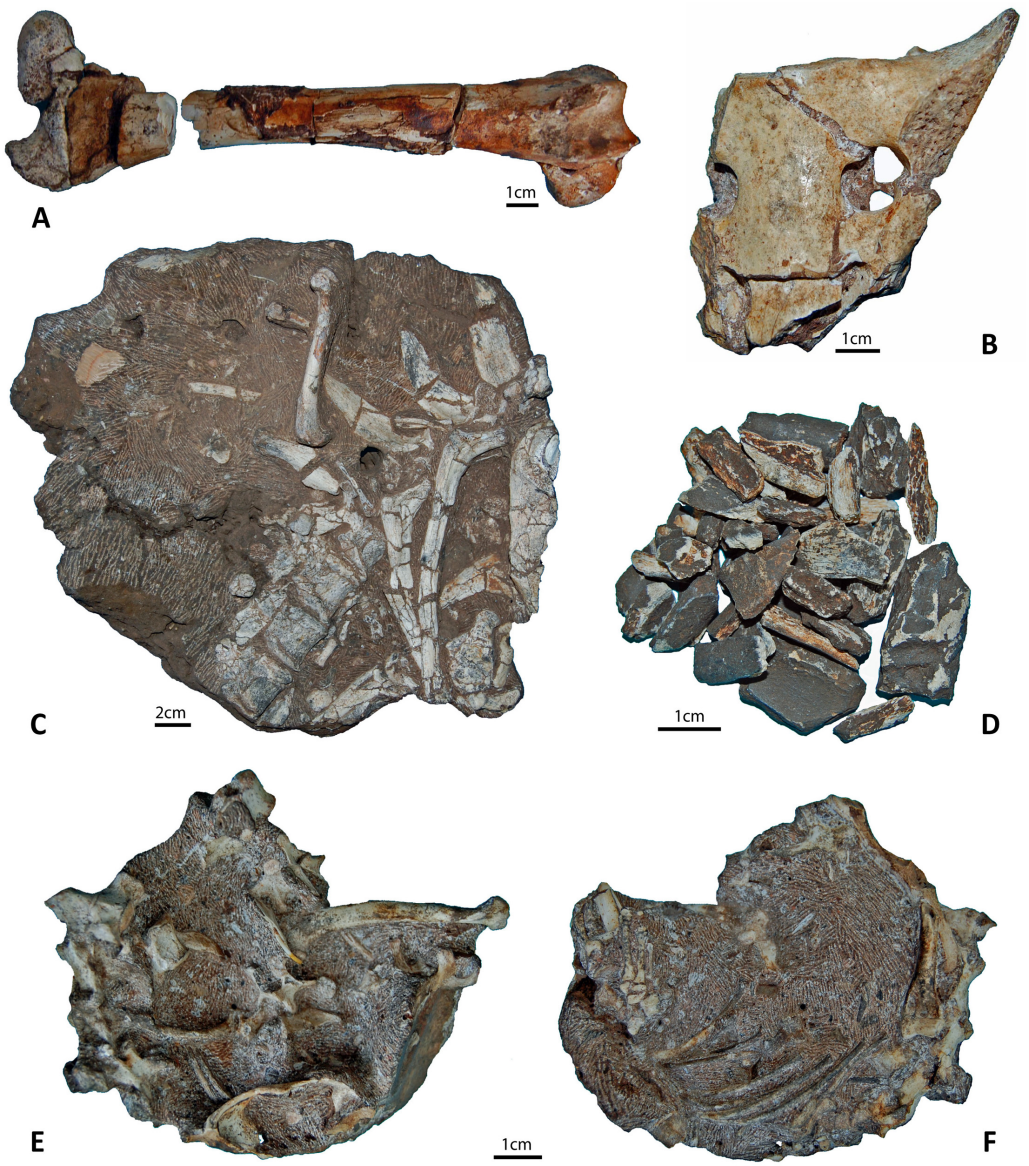
Heterogeneous preservation of fossils in the Malapa faunal assemblage. A) Highly weathered and decalcified bovid femur. B) Unweathered, well-preserved bovid sacrum. C) Articulated bovid ribs and thoracic vertebrae. D) Extremely fragmentary unidentifiable bone fragments recovered from decalcified sediments. E) and F) Superior and inferior views of a complete, articulated, upper skeleton of a small carnivore (*Genetta* sp.).

A few components of the faunal assemblage indicate the introduction of some material from a source area that presents a different taphonomic signature. Apart from well preserved faunal material, fragmentary and poorly preserved bone remains were also recovered (Fig 4). Representing 39.8% of the assemblage (in NISP), these remains are highly weathered (stages 3 to 5; S8 Fig) and have a very invasive manganese coating (Fig 4; S6–S7 Figs). Decalcification appears to have played an important role in the preservation of these fossils; the majority (86.6%) of poorly preserved specimens were recovered from decalcified sediments, while well preserved fossils come from calcified clastic sediments (S6–S7 Figs; S5–S6 Tables).

## Discussion

The results of the analysis of the Malapa faunal assemblage have several implications, which are discussed here. Firstly, based on the taphonomic characteristics of the faunal remains, we propose a hypothetical scenario explaining the modes of accumulation of animal remains inside the cave and subsequent diagenetic processes, namely a natural death trap scenario followed by the action of a debris flow. Hypotheses regarding the possible origin of the unusual abundance of carnivores in the assemblage are also presented, based on comparisons with other fossil death traps from South Africa and elsewhere. Finally, we discuss the significance of the taphonomic specificities observed at Malapa, in the context of Plio-Pleistocene fossil assemblages from the Cradle of Humankind.

### A natural death trap scenario

The abundance of articulated remains, antimeric sets of bones, complete and near complete bones indicates that most of the animals entered the cave system as whole individuals. Several lines of evidence point towards the absence of carnivore contribution to the faunal assemblage. Hence, carnivores known to accumulate bones inside caves (felids and hyaenids) produce predictable skeletal patterns, consistent either with refuse or scat assemblages [1, 42, 43, 52] and associated with the way they consume their prey (e.g. hyaenid preference for spongy epiphyses over hard cortical long bones shafts; or tendency of carnivores to swallow whole small elements, which are consequently found in the form of digested bones inside scat assemblages). On the contrary, the body part representation observed for the fauna at Malapa follows a density-mediated pattern, suggesting that decomposition of carcasses of animals having fallen inside the cave took place without disruption by carnivores. Hyenids are very destructive and tend to produce assemblages with high percentages of broken bones, digested elements and gnawed bones (e.g. bones showing crenulated edges, pits, punctures and furrows) [1, 18, 19, 52]. While being less destructive, leopards also leave gnaw marks and break bones during consumption [1, 18, 19, 48, 52]. At Malapa, the lack of carnivore chewing marks on the faunal remains, together with the abundance of complete and near complete elements and the recovery of elements still in articulation confirm that (1) carnivores did not accumulate carcasses inside the cave; and (2) they did not have access to the cave chamber where carcasses where decomposing. Damage caused by rodents, and especially porcupines (*Hystrix africaeaustralis*), is documented for the majority of Plio-Pleistocene South African fossil assemblages [1, 10, 16]. The absence of rodent damage at Malapa confirms that the cave chamber where animals were decomposing did most likely not offer any other access than the vertical shaft leading to it. Insects and microbes seem to have been the only animals able to reach the carcasses.

The prominence of animals with good climbing proclivities, as is the case at Malapa, is a common feature among faunal assemblages accumulated via natural death traps [17;53–59]. In the Cradle of Humankind, large carnivores and primates dominate the faunal assemblages of Sterkfontein Member 2 (deposit associated with the well preserved australopithecine skeleton StW 573, “Little Foot”) and Peabody’s Pit 23 at Bolt’s Farm, two sites that have yielded fossil assemblages accumulated via a pitfall, and without the contribution of biotic agents [17;53]. In the Holocene assemblage recovered from the pitfall of McEachern’s Deathtrap Cave in Australia, the bones of several arboreal species have been recovered in abundance [56], especially koalas (*Phascolarctos cinereus*) and possums (brush-tailed possum, *Trichosurus vulpecula*; pygmy possum, *Cercatetus nanu*; and ring-tailed possum, *Pseudocheirus peregrinus*), which are considered to be excellent climbers. The presence of leporid remains (*Lepus* sp.) in the Malapa assemblage is interesting because studies of European fossil assemblages accumulated via natural pitfalls have shown the occurrence of hare and rabbit bones to be a constant in such assemblages. In karstic regions, these animals can easily fall inside natural openings while running away from predators [57; 59].

Therefore, based on the composition of the faunal spectrum, body part representations, mortality profiles, breakage patterns, presence of antimeric sets of bones, articulated remains, and lack of gnaw marks on the bones, the contribution of rodents, birds of prey and mammalian predators as major taphonomic agents can be discarded. Rather, the general characteristics of the fossil assemblage point towards a natural death trap scenario [17; 54–59].

In a recent study of landscape dynamics in the Cradle of Humankind [23], the rate of erosion of the African Plateau in the past 4 Millions years was estimated and it was proposed that at the time of the accumulation of the faunal assemblage (ca. 2 Ma BP), the Malapa cave system may have been as deep as 30 m. The presence of articulated remains of large bovids in the assemblage recovered from the pit indicates that the cave entrance must have been large enough to permit animals of this size to enter.

We propose a natural death trap scenario in its wider sense, as defined by Pickering *et al*. [17]. Animals running away from predators or not detecting the opening hidden by vegetation would have fallen directly via a vertical shaft towards a deep part of the cave system offering no access to scavengers—the Malapa site as we know it today. Some animals could have been attracted by water or the smell of decomposing carcasses. Finally, some bones and carcasses could have been directly collected from the surface by gravity or heavy summer rains. Based on the articulated specimens and antimeric sets of bones, we propose that at least seven complete individuals, including the two hominins MH1 and MH2 (Table 1), accumulated inside a cave chamber where they decomposed and mummified.

**Table 1:** Minimum number of individuals represented by complete and near complete skeletons.

Species	Description of the specimens/individuals	MNI
*Au*. *sediba*	MH1 and MH2 (nearly complete individuals)	2
Bovid class II	Various elements in articulation, partial skeleton, 3 antimeric sets of bones; one near complete foetus in articulation	2
Bovid class III (*Tragelaphus* sp.)	Various elements in articulation, partial skeleton, 5 antimeric sets of bones	1
Small carnivore (*Genetta* sp.)	Complete upper body in articulation and near-articulation	1
Lagomorph (*Lepus* sp.)	Articulated bones, 3 antimeric sets of bones	1
**TOTAL**	-	7

The water-laid sediments comprising Facies D are associated with a debris flow event. The occurrence of elements still in articulation or preserved as anatomical proximities consistent with unstable joints (i.e. joints that disarticulate first during the decomposition process) could indicate that some of the decomposing carcasses were either in a relatively fresh state or mummified when the debris flow took place. Conditions inside the dolomitic cave chamber at Malapa (stable temperature and degree of humidity, as well as absence of disturbance by scavengers) were conducive to bone preservation. The occurrence of a few poorly preserved bones mixed with the otherwise very well preserved fossils, is in accordance with the action of a debris flow that mixed bones from various parts of the cave system, most of which accumulated via a natural death trap, and from the surface. All skeletal elements caught in the debris flow would have been transported and buried to a deeper part of the cave system, where they fossilized. Burial by the debris flow created an anaerobic environment allowing for the excellent preservation of the hominin fossils and some of the associated fauna for the next 2 million years.

### Abundance of carnivores in the assemblage

Within extant and extinct faunal communities, respective percentages of carnivores and ungulates are generally disproportionate in favour of ungulates, which are much more abundant in a given ecosystem than their predators. Therefore, a high carnivore/ungulate ratio inside a fossil assemblage, as is the case at Malapa, is likely to be the consequence of some taphonomic bias. In cave contexts, two distinct scenarios can lead to an overrepresentation of carnivore remains within a faunal assemblage: (1) when the cave is used by bone-collecting carnivores such as leopards and hyaenas, and especially by the brown hyaena *P*. *brunnea*, a taxon that includes a high percentage of carnivores in its diet [49]; or (2) when carcasses are naturally accumulated via a pitfall. In the second scenario, carnivores are attracted by the smell of decomposing animals. Endowed with good climbing skills, they might venture inside the cave system and find themselves trapped.

Several examples of fossil natural death trap assemblages characterised by an unusually high percentage of carnivores are documented in the literature. In South Africa, two fossil Plio-Pleistocene deposits in the Cradle of Humankind have also yielded a significant percentage of carnivore remains: Member 2 at Sterkfontein, associated with Stw 573; and Peabody’s Pit 23 at Bolt’s Farm. In both cases, the fauna is dominated by felids and cercopithecoids. At Sterkfontein Member 2, felids are represented by the leopard *Panthera pardus* (MNI: 2), the lion *P*. *leo* (MNI: 1) and the caracal *Felis caracal* (MNI: 4) [17]. As in the case of Malapa, the fauna is characterised by the presence of antimeric sets of bones, articulated elements, partial skeletons and rare indications of carnivore gnawing. The faunal assemblage from Peabody’s Pit 23 in Bolt’s Farm contains well preserved cranial associated with post-cranial material preserving some limb elements in partial articulation, belonging to three *Dinofelis barlowi* individuals (two adults and one juvenile), considered to be part of the same family [53]. For both assemblages, the hypothesis of the use of the cave by the carnivores as a den has been ruled out, based, for Sterkfontein Member 2, on the low degree of carnivore damage on the bones, the absence of digested bones, coprolites and juvenile carnivore remains [17]; and for Peabody’s Pit 23, on the absence of ungulate remains in the assemblage [53]. Rather, the authors suggested that the carnivores intentionally entered the cave, which was probably difficult to access, attracted by water or decomposing animals, and were unable to get out. Other examples of unusually high percentages of carnivores inside fossil assemblages are also documented outside of Africa. Hence, at Natural Trap Cave (Wyoming, USA) and at l’Igue du Gral (Lot, France), gray wolves (*Canis lupus*) are remarkably abundant [54; 59]. At Coudoulous, remains of canids and hyaenids, namely gray wolves and European cave hyaenas (*Crocuta spelaea*), have also been collected in significant quantities [55]. In these three cases, it has been suggested that the carnivores, attracted by animals decomposing inside the cave system, tried to climb down and died, either as a direct consequence of the fall, or due to thirst and starvation.

### Malapa: a unique case in the Cradle of Humankind

The results of this study confirm the complexity of the interactions between the taphonomic processes, which have contributed to the accumulation and modification of bones in the different caves from the Cradle of Humankind. As pointed out by Adams [10], each South African fossil assemblage has a unique taphonomic and ecological history that influenced the composition of the assemblage [10]. The fauna from Malapa constitutes no exception to the rule. The assemblage is the result of a unique combination of taphonomic processes, which include the capture of animals via a vertical shaft in a part of the cave system offering limited, if any, access to mammalian scavengers, surface modification of bone by invertebrates, and the action of a debris flow, which led to the remarkable state of preservation of the hominins and some of the associated fauna.

Fossil remains recovered from cave deposits in the Cradle of Humankind are usually isolated, fragmentary and poorly preserved (e.g. weathered, having suffered from decalcification, covered with manganese). This is especially true of hominin remains, which are normally not only poorly preserved, but also very rare. Together with StW 573 and the fauna associated with it in Sterkfontein Member 2 [14, 15, 17], Malapa constitutes, in terms of preservation, a unique case in the context of Plio-Pleistocene cave deposits in the Bloubank Valley. Besides the exceptional degree of bone preservation, another remarkable feature of the assemblage is the significant number of hominin remains; a quarter of the faunal assemblage. Another notable difference between Malapa and the other South African sites considered to have accumulated via a natural death trap is the almost complete absence of non-hominin primates. Cercopithecoids at Malapa are represented by a single skull attributed to *Papio* sp. [40], while remains of *Papio*, *Parapapio* and *Cercopithecoides williamsi* are abundant at Peabody Pit 23 in Bolt’s Farm and Sterkfontein Member 2. The recovery of more material during future excavations should enable us to test whether this is due to sampling bias, or is the reflection of a genuine rarity of cercopithecoids in the Malapa assemblage, possibly related to territorial behaviour [60].

## Conclusion

A natural death trap scenario combined with debris flow is the most parsimonious explanation for the accumulation and fossilization of the faunal material recovered in association with the two nearly complete *Au*. *sediba* skeletons (MH1 and MH2) from Malapa. A thorough taphonomic analysis confirmed the introduction of a large proportion of the animals in the deposit via a vertical shaft leading to a part of the cave that offered no access to mammalian carnivores, birds of prey or rodents. A debris flow incorporated skeletons in that part of the cave, which, based on their state of articulation and completeness, were likely mummified. This debris flow collected elements from other parts of the karstic system, and probably from the surface, before settling in a lower part of the cave system and lithifying to form breccia. This combination of taphonomic events is apparently unique in the context of cave sites in the Cradle of Humankind, and contributes to explaining the remarkable state of preservation of the hominins and some of the associated fauna.

## Supporting Information

S1 Dataset Complete excel database about the faunal assemblage from Malapa, including taxonomic, anatomical and taphonomic descriptions. (XLSX)

S1 Fig Skeletal body part survival percentages for ungulates and carnivores. (TIF)

S2 Fig Examples of articulated non-hominin faunal remains in blocks. A) One bovid femur, two tibiae, and one talus in block UW88-B848. B) Bovid thoracic vertebrae associated with bovid ribs, one humerus and an ungulate mandible with teeth, in block UW88-B375. C) Bovid humerus articulated with a radio-ulna, in block UW88-B051.
(TIF)

S3 Fig Examples of non-hominin faunal remains in near articulation, still embedded in calcified sediment. A) Mammal ribs in block UW88-B1043. B) Bovid humerus and associated scapula in block UW88-B243. C) Bovid ribs in block UW88-B152.
(TIF)

S4 Fig Examples of non-hominin faunal remains in anatomical proximity. A) Bovid left ankle (UW88-1156 to 1160). B) Large bovid carpals (UW88-1259a to 1259c). C) Bovid atlas, axis and third cervical vertebra (UW88-720-722). D) Hyaenid phalanges (UW88-782 and 783). E) Bovid ribs articulated with a thoracic vertebra (no specimen number). F) Rodent skull and associated mandible (UW88-781).
(TIF)

S5 Fig Mortality profile observed at Malapa, combining age estimates for the hominins, carnivores and ungulates. (TIF)

S6 Fig Different degrees of manganese coating observed on the bones. From left to right: slight, slight to moderate, moderate, moderate to heavy, heavy.
(TIF)

S7 Fig Comparison of degree of manganese dioxide perimineralization according to the provenance of the remains (decalcified sediment, in orange, versus calcified sediment, in light pink). (TIF)

S8 Fig Comparison of degree of weathering according to the provenance of the remains (decalcified sediment, in dark green, versus calcified sediment, in light green). (TIF)

S1 Table Quantitative data for the bovid and carnivore remains used to calculate the percentage of survival for each anatomical element for both groups. (DOCX)

S2 Table List of non-hominin faunal remains recovered in articulation. (DOCX)

S3 Table List of non-hominin faunal specimens recovered as anatomical proximities. (DOCX)

S4 Table List of antimeric sets of bones present in the Malapa non-hominin faunal assemblage. (DOCX)

S5 Table Comparison of degree of manganese dioxide coating according to the provenance of the remains (decalcified versus calcified sediment). (DOCX)

S6 Table Weathering stages observed in the non-hominin faunal assemblage. (DOCX)

## References

[1] Brain CK. The hunters or the hunted? Introduction to African cave taphonomy. Chicago: The University of Chicago Press; 1981.

[2] Grine FE. New hominid fossils from the Swartkrans Formation (1979–1986 excavations): craniodental specimens. Am J Phys Anthropol. 1989; 79: 409–449. 2672828 10.1002/ajpa.1330790402

[3] Grine FE. Description and preliminary analysis of new hominid craniodental fossils from the Swartkrans Formation In: Brain CK, editor. Swartkrans: a cave’s chronicle of early man. Pretoria: Transvaal Museum Monograph no.8; 1993 pp. 75–116. 10.1038/nature10149

[4] Watson V. Composition of the Swartkrans bone accumulations, in terms of skeletal parts and animals represented In: Brain CK, editor. Swartkrans, a cave’s chronicle of early man. Pretoria: Transvaal Museum Monograph no.8; 1993 pp. 35–73. 10.1038/nature10149

[5] Keyser AW, Menter CG, Moggi-Cecchi J, Pickering TR, Berger LR. Drimolen: a new hominid-bearing site in Gauteng, South Africa. South Afr J Sci. 2000; 96: 193–197.

[6] Kuman K, Clarke RJ. Stratigraphy, artefact industries and hominid associations for Sterkfontein, Member 5. J Hum Evol. 2000; 38: 827–847. 10835264 10.1006/jhev.1999.0392

[7] Tobias PV. The Fossil Hominids In: Partridge TC, Maud RR, editors. The Cenozoic of Southern Africa. Oxford University Press: Oxford Monographs on Geology and Geophysics, no.40; 2000 pp. 252–276.

[8] Adams JW. Taphonomy and Palaeoecology of the Gondolin Plio-Pleistocene cave site, South Africa. Unpublished PhD Dissertation, University of the Witwatersrand: Johannesburg. 2006.

[9] Adams JW, Herries AIR, Kuykendall KL, Conroy GC. Taphonomy of a South African cave: geological and hydrological influences on the GD 1 fossil assemblage at Gondolin, a Plio-Pleistocene paleocave system in the Northwest Province, South Africa. Quat Sci Rev. 2007a; 26: 2526–2543.

[10] Adams JW, Hemingway J, Kegley ADT, Thackeray JF. Luleche, a new paleontological site in the Cradle of Humankind, North-West Province, South Africa. J Hum Evol. 2007b; 53: 751–754. 17935756 10.1016/j.jhevol.2007.08.009

[11] Gommery D, Sénégas F, Thackeray JF, Potze S, Kgasi L, Claude J, et al Plio-Pleistocene fossils from Femur Dump, Bolt’s Farm, Cradle of Humankind World Heritage Site. Annals of the Transvaal Museum 2008; 45: 67–76.

[12] de Ruiter DJ, Pickering R, Steininger CM, Kramers JD, Hancox PJ, Churchill SE, et al New *Australopithecus robustus* fossils and associated U-Pb dates from Cooper’s Cave (Gauteng, South Africa). J Hum Evol. 2009; 56: 497–513. 10.1016/j.jhevol.2009.01.009 19443017

[13] Reynolds SC, Kibii JM. Sterkfontein at 75: review of palaeoenvironments, fauna and archaeology from the hominin site of Sterkfontein (Gauteng Province, South Africa). Palaeontologia Africana 2011; 46: 59–88.

[14] Clarke RJ. The first ever discovery of a well-preserved skull and associated skeleton of *Australopithecus* . South Afr J Sci. 1998; 94: 460–463.

[15] Clarke RJ. Latest information on Sterkfontein’s *Australopithecus* skeleton and a new look at *Australopithecus* . South Afr J Sci. 2008; 104: 443–449.

[16] Brain CK. A taphonomic overview of the Swartkrans fossil assemblages In: Brain CK, editor. Swartkrans, a cave’s chronicle of early man. Pretoria: Transvaal Museum Monograph no.8; 1993 pp. 257–264. 10.1038/nature10149

[17] Pickering TR, Clarke RJ, Heaton JL. The context of Stw 573, an early hominid skull and skeleton from Sterkfontein Member 2: taphonomy and paleoenvironment. J Hum Evol. 2004a; 46: 279–297. 14984784 10.1016/j.jhevol.2003.12.001

[18] Pickering TR, Clarke RJ, Moggi-Cecchi J. The role of carnivores in the accumulation of the Sterkfontein Member 4 hominid assemblage: a taphonomic reassessment of the complete hominid fossil sample (1936–1999). Am J Phys Anthropol. 2004b; 125:1–15. 15293327 10.1002/ajpa.10278

[19] Pickering TR, Domínguez-Rodrigo M, Egeland CP, Brain CK. Beyond leopards: tooth marks and the contribution of multiple carnivore taxa to the accumulation of the Swartkrans Member 3 fossil assemblage. J Hum Evol. 2004c; 46: 595–604. 15120267 10.1016/j.jhevol.2004.03.002

[20] Clarke RJ. Taphonomy of Sterkfontein *Australopithecus* skeletons In: Pickering TR, Schick K, Toth N, editors. Breathing life into fossils: taphonomic studies in honor of C.K. (Bob) Brain. Bloomington (Indiana): Stone Age Institute Press; 2007 pp. 199–205.

[21] Berger LR, de Ruiter DJ, Churchill SE, Schmid P, Carlson KJ, Dirks PHGM, et al *Australopithecus sediba*, a new species of *Homo*-like Australopith from South Africa. Science 2010; 328: 195–204. 10.1126/science.1184944 20378811

[22] Dirks PHGM, Kibii JM, Kuhn BF, Steininger C, Churchill SE, Kramers JD, et al Geological setting and age of *Australopithecus sediba* from Southern Africa. Science 2010; 328: 205–208. 10.1126/science.1184950 20378812

[23] Dirks PHGM, Berger LR. Hominin-bearing caves and landscape dynamics in the Cradle of Humankind, South Africa. J Afr Earth Sci. 2013; 78: 109–131.

[24] Berger LR. *Australopithecus sediba* and the earliest origins of the genus *Homo* . J Anthropol Sci. 2012; 90: 1–16. 10.4436/jass.90009 23011933

[25] Pickering R, Dirks PHGM, Jinnah Z, de Ruiter DJ, Churchill SE, Herries AIR, et al *Australopithecus sediba* at 1.977 Ma and implications for the origins of the genus *Homo* . Science 2011; 333: 1421–1423. 10.1126/science.1203697 21903808

[26] Payne S. Partial recovery and sample bias In: Clason AT, editor. Archaeozoological studies. Amsterdam: North Holland; 1975 pp. 27–17.

[27] Lyman RL. Bone density and differential survivorship of fossil classes. J Anthropol Archaeol. 1984; 3: 259–299.

[28] Klein RG, Cruz-Uribe K. The analysis of animal bones from archaeological sites. Chicago: University of Chicago Press; 1984.

[29] Davis SJM. The archaeology of animals. New Haven: Yale University Press; 1987.

[30] Lyman RL. Vertebrate taphonomy. Cambridge University Press: Cambridge Manuals in Archaeology; 1994.

[31] Bunn HT. Meat-eating and human evolution: studies on the diet and subsistence patterns of Plio-Pleistocene hominids in East Africa. Ph.D. Thesis, University of California at Berkeley. 1982.

[32] Bunn HT, Kroll EM, Ambrose SH, Behrensmeyer AK, Binford LW, Blumenschine RJ, et al Systematic butchery by Plio-Pleistocene hominids at Olduvai Gorge, Tanzania. Curr Anthropol. 1986; 27: 431–452.

[33] Plug C, Plug I. MNI counts as estimates of species abundance. South Afr Archaeol Bull. 1990; 45(151): 53–57.

[34] Brain CK. The contribution of Namib desert Hottentots to an understanding of australopithecine bone accumulations. Scientific Papers of the Namib Desert Research Station 1969; 39: 13–22.

[35] Brain CK. Some principles in the interpretation of bone accumulations associated with Man In: Isaac GL, McCown ER, editors. Human origins: Louis Leakey and the East African evidence. California: W. A. Benjamin, Menlo Park; 1976 pp. 97–116.

[36] Lyman RL. Quantitative units and terminology in zooarchaeology. Am Antiq. 1994; 59(1): 36–71.

[37] Villa P, Mahieu E. Breakage patterns of human long bones. J Hum Evol. 1991; 21: 27–48.

[38] Kuhn BF, Werdelin L, Hartstone-Rose A, Lacruz RS, Berger LR. Carnivoran remains from the Malapa hominin site, South Africa. Plos ONE 2011; 6(11) e26940 10.1371/journal.pone.0026940 22073222 PMC3207828

[39] Val A, Carlson KJ, Steininger C, Kibii JM, Churms C, Kuhn BF, et al 3D techniques and fossil identification: an elephant shrew mandible from the Malapa site. South Afr J Sci. 2011; 107: 1–5.

[40] Gilbert CC, Steininger CM, Kibii JM, Berger LR (submitted) A *Papio* cranium from Malapa: implications for the evolution of modern baboon cranial morphology and South African Plio-Pleistocene biochronology. 10.1371/journal.pone.0133361 PMC4545885 26287673

[41] Lyman RL. On the analysis of vertebrate mortality profiles: sample size, mortality type, and hunting pressure. Am Antiq. 1987; 52: 125–142.

[42] Carlson KJ, Pickering TR. Intrinsic qualities of primate bones as predictors of skeletal element representation in modern and fossil carnivore feeding assemblages. J Hum Evol. 2003; 44: 431–450. 12727462 10.1016/s0047-2484(03)00025-3

[43] Pickering TR, Carlson KJ. Baboon taphonomy and its relevance to the investigation of large felid involvement in human forensic cases. For Sci Int. 2004; 144: 37–44. 15240019 10.1016/j.forsciint.2004.03.003

[44] Lyman RL. Bone density and differential survivorship of fossil classes. J Anthropol Archaeol. 1984; 3: 259–299.

[45] Kreutzer LA. Bison and deer bone mineral densities: comparisons and implications for the interpretation of archaeological faunas. J Archaeol Sci. 1992; 19: 271–294.

[46] Lam YM, Chen X, Pearson OM. Intertaxonomic variability in patterns of bone density and the differential representation of bovid, cervid, and equid elements in the archaeological record. Am Antiq. 1999; 64: 343–362.

[47] Novecosky BJ, Popkin PRW. Canidae volume bone mineral density values: an application to sites in western Canada. J Archaeol Sci. 2005; 32: 1677–1690.

[48] de Ruiter DJ, Berger LR. Leopards as taphonomic agents in dolomitic caves—implications for bone accumulation in the hominid-bearing deposits of South Africa. J Archaeol Sci. 2000; 27: 665–684.

[49] Skinner JD, van Aarde RJ. Bone collecting by brown hyaenas *Hyaena brunnea* in the Central Namib Desert, Namibia. J Archaeol Sci. 1991; 18: 513–523.

[50] Marean CW, Ehrhardt CL. Paleoanthropological and paleoecological implications of the taphonomy of a sabertooth’s den. J Hum Evol. 1995; 29: 515–547.

[51] Behrensmeyer AK. Taphonomic and ecologic information from bone weathering. Paleobiol. 1978; 4: 150–162.

[52] Domínguez-Rodrigo M, Pickering TR. A multivariate approach for discriminating bone accumulations created by spotted hyenas and leopards: harnessing actualistic data from East and Southern Africa. J Taphonomy 2010; 8(1–2): 155–179.

[53] Cooke HBS. *Dinofelis barlowi* (Mammalia, Carnivore, Felidae) cranial material from Bolt’s Farm, collected by the University of California African expedition. Palaeontol Afr. 1991; 28: 9–21.

[54] Wang X, Martin LD. Late Pleistocene, paleoecology and large mammal taphonomy, Natural Trap Cave, Wyoming. Nat Geogr Res Explor. 1993; 9(4): 422–435

[55] Costamagno S. Coudoulous II: taphonomie d’un aven-piège. Contribution des accumulations d’origine naturelle à l’interprétation des archéofaunes du Paléolithique moyen. Anthropozoologica 1999; 29: 13–31.

[56] Kos AM. Characterisation of post-depositional taphonomic processes in the accumulation of mammals in a pitfall cave deposit from southeastern Australia. J Archaeol Sci. 2003; 30: 781–796.

[57] Coumont MP. Taphonomie préhistorique: mammifères fossiles en contexte naturel, les avens-pièges, apport pour l’étude des archéofaunes. Unpublished PhD Dissertation: Université Aix-Marseille, France. 2006.

[58] Coumont MP. Proposition d’un référentiel taphonomique fossile de faunes issues d’avens-pièges. Annales de Paléontologie 2009; 95: 1–20.

[59] Castel JC, Coumont MP, Boudadi-Maligne M, Prucca A. Rôle et origine des grands carnivores dans les accumulations naturelles. Le cas des loups (*Canis lupus*) de l’Igue du Gral (Sauliac-sur-Célé, Lot, France). Rev Paleobiol Genève 2010; 29(2): 411–425.

[60] Val A, Taru P, Steininger C. New taphonomic analysis of large-bodied primate assemblage from Cooper’s D, Bloubank Valley, South Africa. South Afr Archaeol Bull. 2014; 69(199): 49–58.

