# Supplementary figures and images for: Taphonomic Analysis of the Faunal Assemblage Associated with the Hominins (Australopithecus sediba) from the Early Pleistocene Cave Deposits of Malapa, South Africa
Source: PLoS One. 2015 Jun 10;10(6):e0126904. doi: 10.1371/journal.pone.0126904 (PMC4465193; doi:10.1371/journal.pone.0126904)

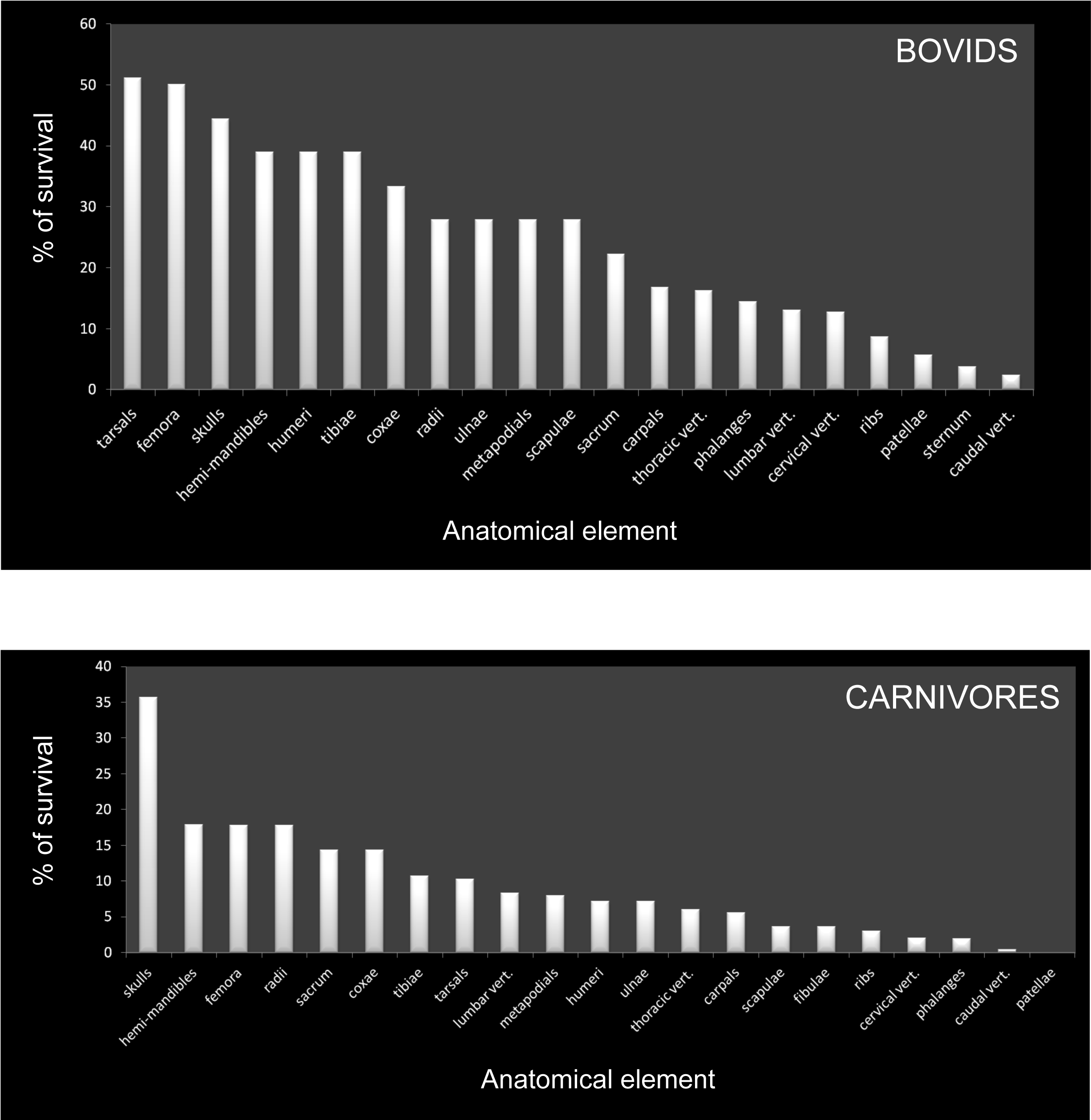

Supplement: S1 Fig — (TIF) [file pone.0126904.s002.tif]

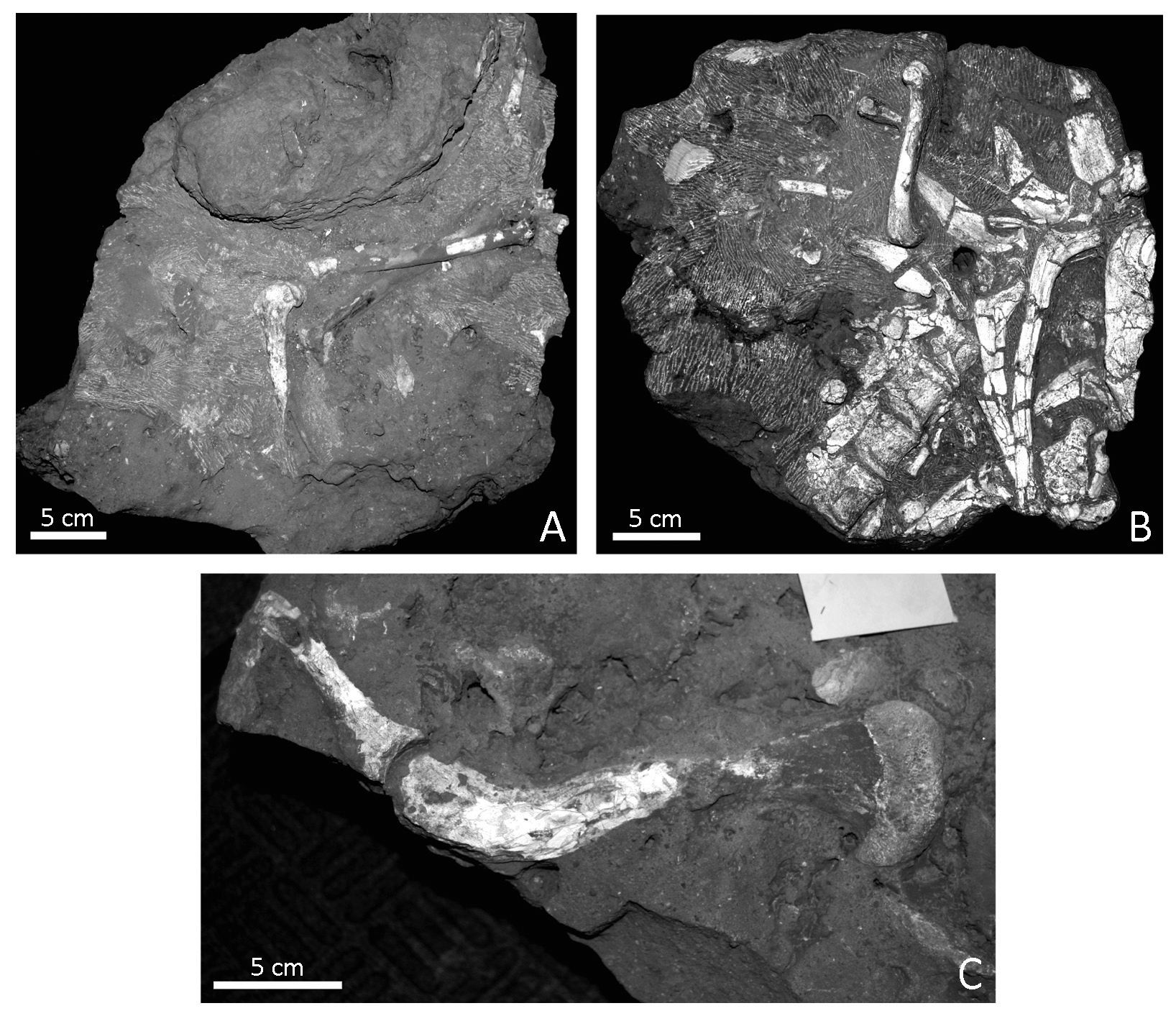

Supplement: S2 Fig — A) One bovid femur, two tibiae, and one talus in block UW88-B848. B) Bovid thoracic vertebrae associated with bovid ribs, one humerus and an ungulate mandible with teeth, in block UW88-B375. C) Bovid humerus articulated with a radio-ulna, in block UW88-B051. (TIF) [file pone.0126904.s003.tif]

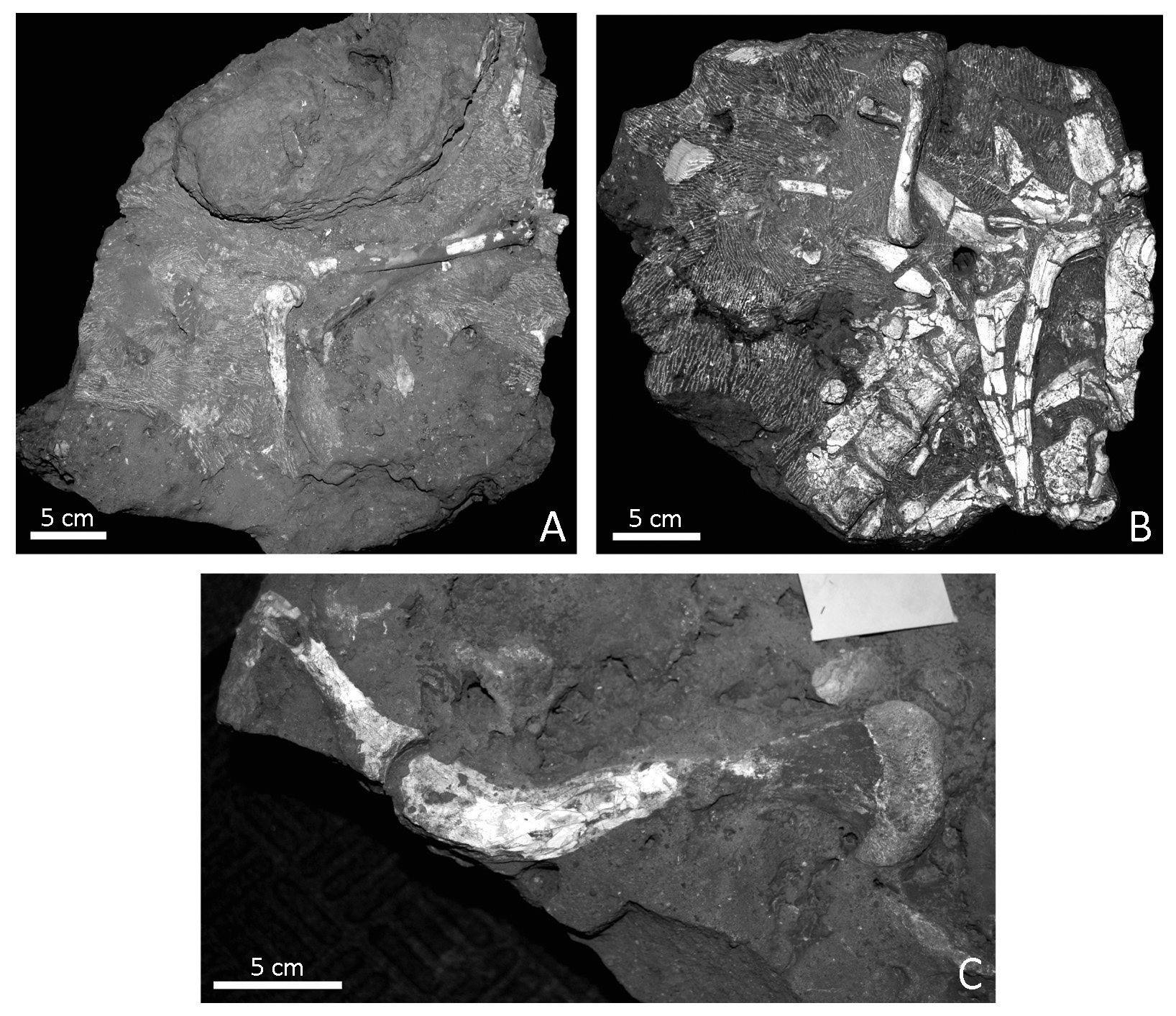

Supplement: S3 Fig — A) Mammal ribs in block UW88-B1043. B) Bovid humerus and associated scapula in block UW88-B243. C) Bovid ribs in block UW88-B152. (TIF) [file pone.0126904.s004.tif]

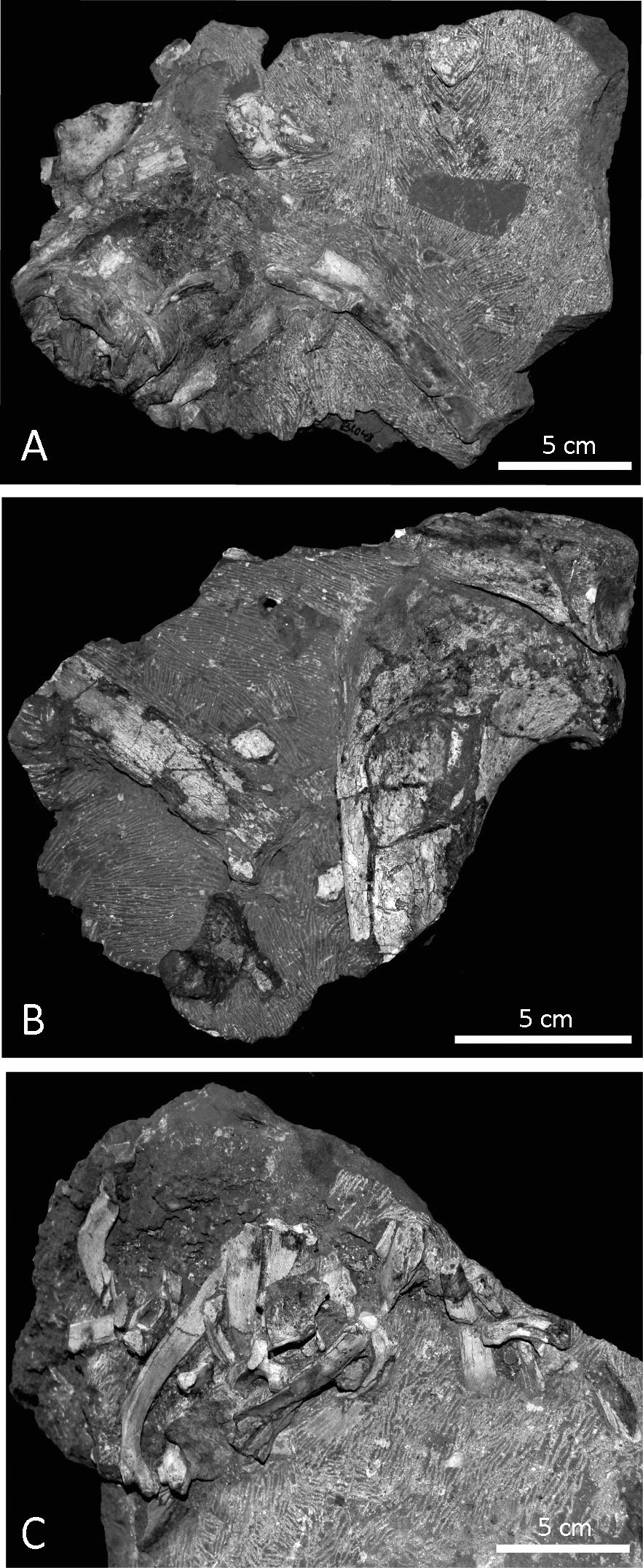

Supplement: S4 Fig — A) Bovid left ankle (UW88-1156 to 1160). B) Large bovid carpals (UW88-1259a to 1259c). C) Bovid atlas, axis and third cervical vertebra (UW88-720-722). D) Hyaenid phalanges (UW88-782 and 783). E) Bovid ribs articulated with a thoracic vertebra (no specimen number). F) Rodent skull and associated mandible (UW88-781). (TIF) [file pone.0126904.s005.tif]

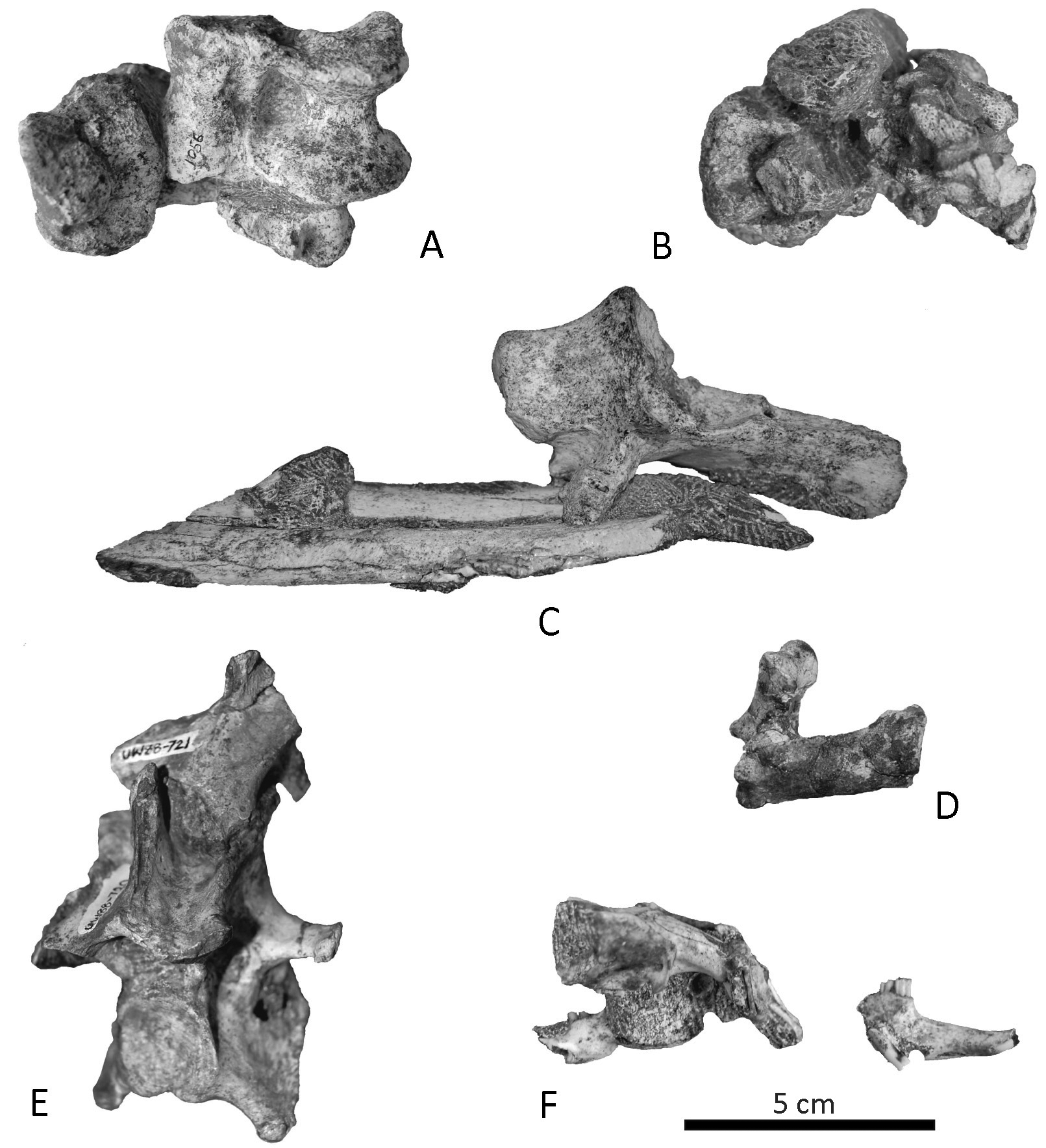

Supplement: S5 Fig — (TIF) [file pone.0126904.s006.tif]

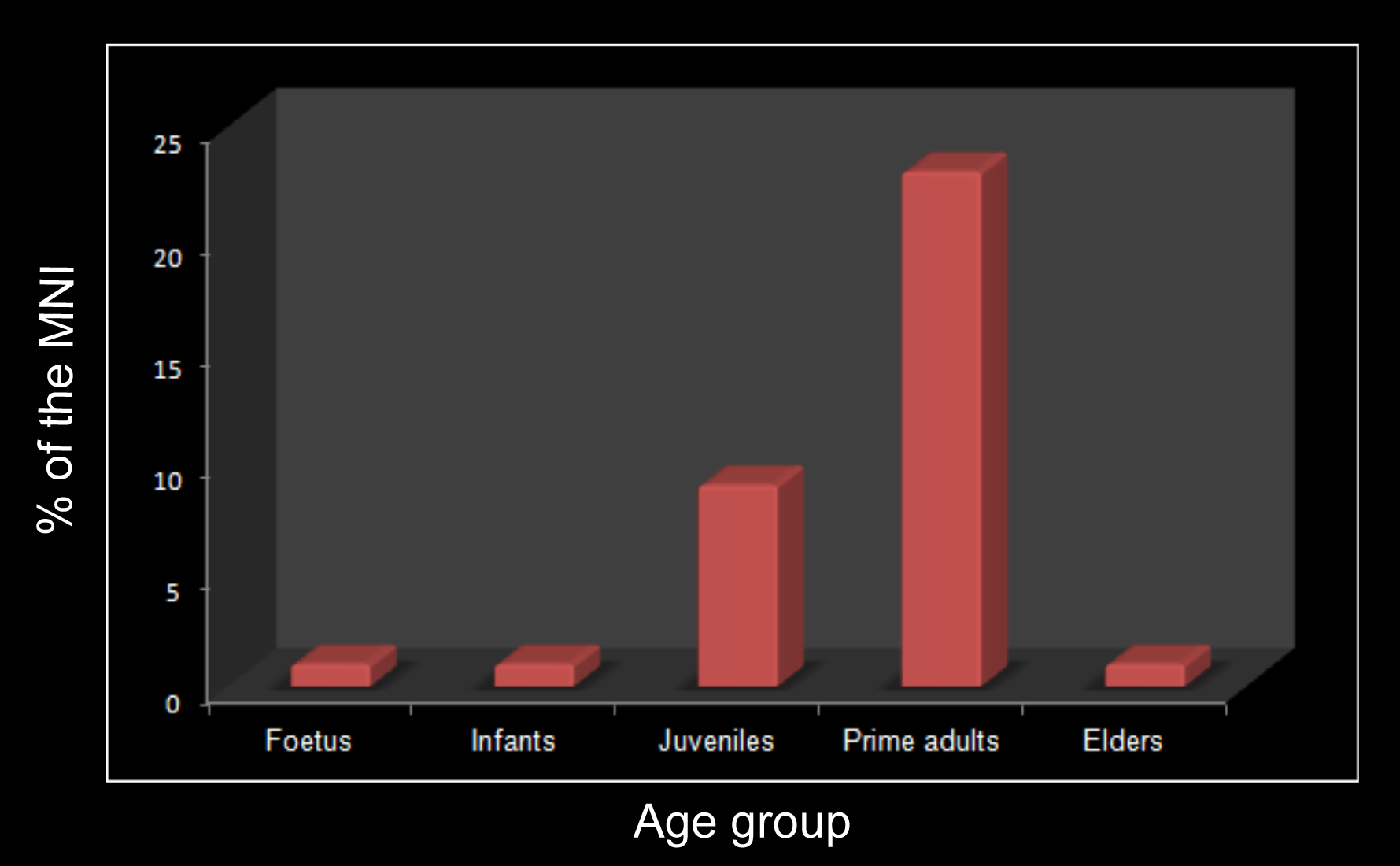

Supplement: S6 Fig — From left to right: slight, slight to moderate, moderate, moderate to heavy, heavy. (TIF) [file pone.0126904.s007.tif]

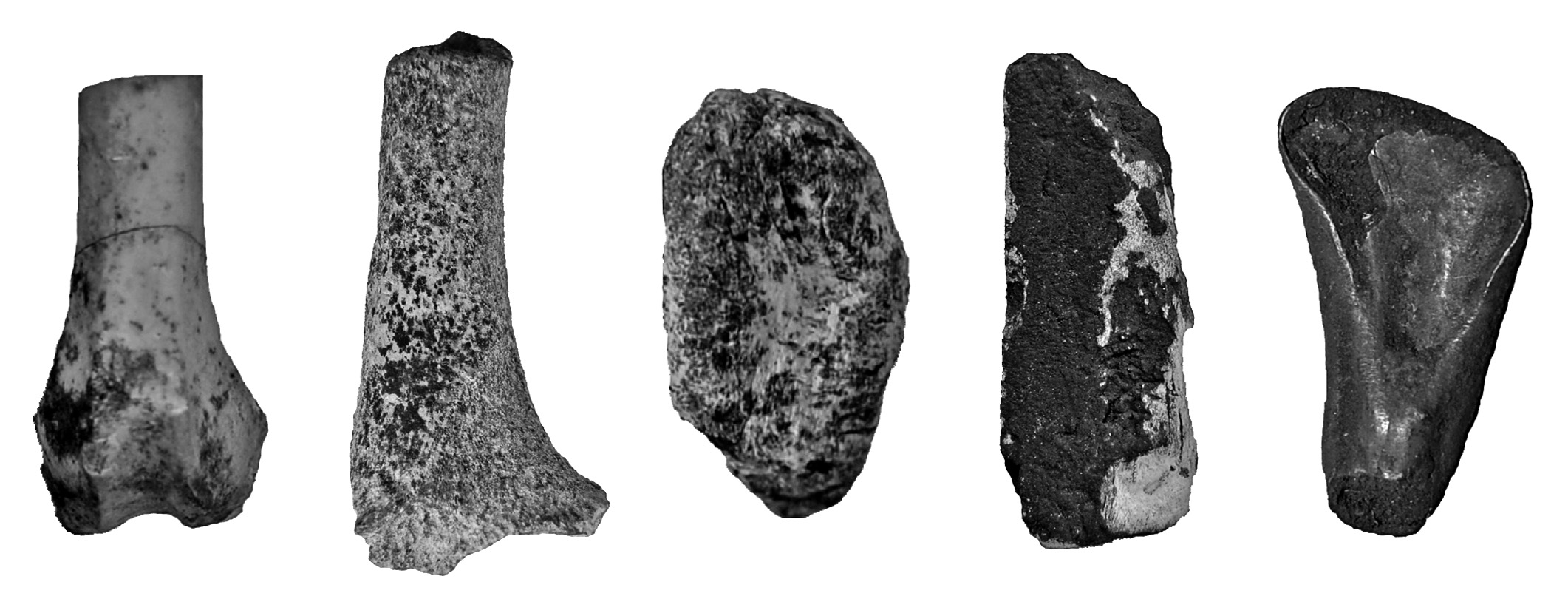

Supplement: S7 Fig — (TIF) [file pone.0126904.s008.tif]

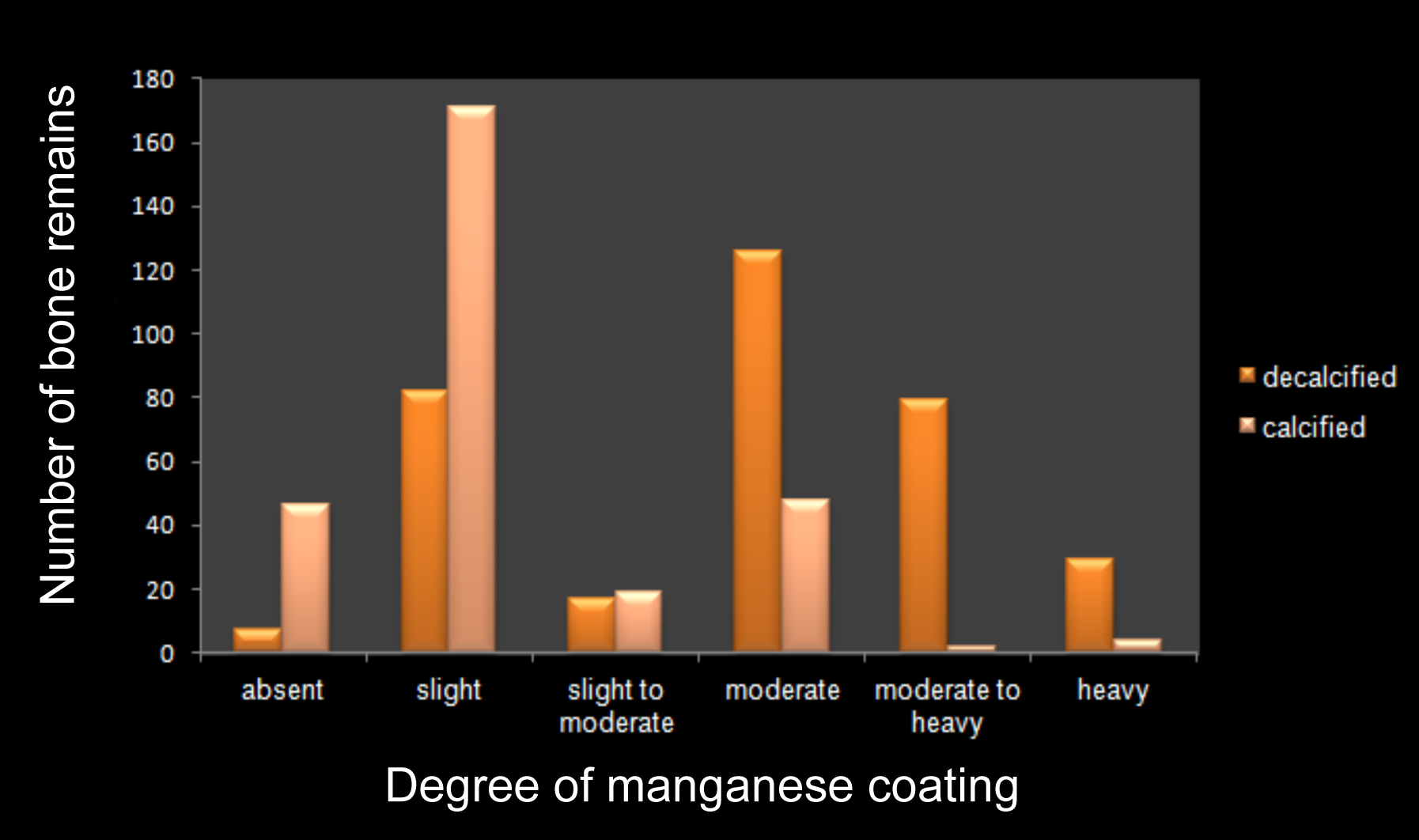

Supplement: S8 Fig — (TIF) [file pone.0126904.s009.tif]
