# Supplementary material for: Taphonomic Analysis of the Faunal Assemblage Associated with the Hominins (Australopithecus sediba) from the Early Pleistocene Cave Deposits of Malapa, South Africa
Source: PLoS One. 2015 Jun 10;10(6):e0126904. doi: 10.1371/journal.pone.0126904 (PMC4465193; doi:10.1371/journal.pone.0126904)
Supplement: S1 Table — (DOCX) [file pone.0126904.s010.docx]

**Table S1.**

|  | N* | NISP | MNE | % survival |
| --- | --- | --- | --- | --- |
| Skulls | 1 | 16/6 | 4/5 | 44.4/35.7 |
| hemi-mandibles | 2 | 10/7 | 7/5 | 38.9/17.9 |
| Sternum | 3/0 | 1/- | 1/- | 3.7/- |
| Ribs | 26 | 34/11 | 20/11 | 8.6/3.0 |
| Cervical vertebrae | 7 | 8/2 | 8/2 | 12.7/2 |
| Thoracic vertebrae | 13 | 20/11 | 19/11 | 16.2/6 |
| Lumbar vertebrae | 6 | 10/7 | 7/7 | 13/8.3 |
| Caudal vertebrae | 19 | 4/1 | 4/1 | 2.3/0.4 |
| Sacrum | 1 | 2/2 | 2/2 | 22.2/14.3 |
| Coxae | 2 | 11/4 | 6/4 | 33.3/14.3 |
| Scapulae | 2 | 9/1 | 5/1 | 27.8/3.6 |
| Humeri | 2 | 9/2 | 7/2 | 38.9/7.1 |
| Radii | 2 | 9/5 | 5/5 | 27.8/17.8 |
| Ulnae | 2 | 5/2 | 5/2 | 27.8/7.1 |
| Femora | 2 | 16/6 | 9/5 | 50/17.8 |
| Patellae | 2 | 1/0 | 1/0 | 5.6/0 |
| Tibiae | 2 | 7/3 | 7/3 | 38.9/10.7 |
| Lateral malleolus/fibula | 2 | -/1 | -/1 | -/3.8 |
| Carpals | 12/14 | 9/5 | 9/5 | 16.7/2.7 |
| Tarsals | 10/14 | 23/10 | 23/10 | 51.1/10.2 |
| Metapodials | 4 | 24/21 | 10/20 | 27.8/7.9 |
| Phalanges | 24/52 | 38/14 | 31/14 | 14.4/1.9 |

*N: number of elements in a complete skeleton.

N.B. data are given respectively for bovids and carnivores.

NB: scapulae are represented almost exclusively by fragments of glenoid cavities; and pelvises by acetabulum and ilium fragments.
