# Supplementary material for: Taphonomic Analysis of the Faunal Assemblage Associated with the Hominins (Australopithecus sediba) from the Early Pleistocene Cave Deposits of Malapa, South Africa
Source: PLoS One. 2015 Jun 10;10(6):e0126904. doi: 10.1371/journal.pone.0126904 (PMC4465193; doi:10.1371/journal.pone.0126904)
Supplement: S2 Table — (DOCX) [file pone.0126904.s011.docx]

**Table S2**.

| Taxon | Specimen number | Articulation |
| --- | --- | --- |
| Brown hyaena | UW88-539 | Ankle |
| Large felid | UW88-747 | Ankle |
| Bovid | UW88-650 | Foot (two articulated proximal phalanges, two intermediate phalanges, two distal phalanges and two sesamoids) |
| Bovid | UW88-751 to 756 | Foot (one metatarsal, a proximal phalanx and four sesamoids) |
| Bovid | UW88-528 | An intermediate phalanx, a distal phalanx and one sesamoid |
| Bovid | no number* | An intermediate phalanx and a distal phalanx |
| Bovid | no number* | Nine thoracic vertebrae |
| Bovid | no number* | Femur articulated with a tibia |
| Bovid | no number* | Humerus articulated with a radio-ulna |
| bovid | no number* | Near complete foetus in articulation |
| Equid | UW88 548-549-550 | Carpals (unciform, accessory metacarpal and magnum) |
| Leporid | UW88-769 | Sacrum articulated with the last 3 lumbar vertebrae |

*: fossils still encased in calcified sediment and awaiting manual preparation, which have not been attributed a specimen number yet.
