# Supplementary material for: Taphonomic Analysis of the Faunal Assemblage Associated with the Hominins (Australopithecus sediba) from the Early Pleistocene Cave Deposits of Malapa, South Africa
Source: PLoS One. 2015 Jun 10;10(6):e0126904. doi: 10.1371/journal.pone.0126904 (PMC4465193; doi:10.1371/journal.pone.0126904)
Supplement: S3 Table — (DOCX) [file pone.0126904.s012.docx]

**Table S3.**

| Taxon | Specimen number | Anatomical proximity |
| --- | --- | --- |
| Bovid | UW88-1156 to 1160 | Ankle (talus, calcaneum, lateral malleolus, cuneiform and cubo-navicular) |
| Bovid | UW88-1259a, b and c | Carpals (fragmentary scaphoid, hamate and magnum) |
| Bovid | no number* | Humerus and scapula |
| Bovid | no number* | ribs |
| Bovid | UW88-720 and 721 | Two cervical vertebrae |
| Bovid | no number* | Three cervical vertebrae (atlas, axis and third cervical vertebra) |
| Bovid | no number* | Two ribs and a thoracic vertebra |
| Bovid | no number* | Elements of a left lower limb (tibia, metatarsal, talus, cubo-navicular) |
| Rodent | UW88-781 | Skull and associate mandible |
| Small carnivore (*Genetta* sp.) |  | Complete upper body (hemi-mandible, left zygomatic and associated teeth, right and left humeri, right scapula, radius and ulna, four right metacarpals, from rank II to V, two carpals, ten ribs, ten thoracic vertebra, six lumbar vertebra and a sacrum). |
| Hyaena | UW88-782 and 783 | Phalanges (one proximal and one intermediate) |
| Mammal | no number* | ribs |

*: fossils still encased in calcified sediment and awaiting manual preparation, which have not been attributed a specimen number yet.
