# Supplementary material for: Taphonomic Analysis of the Faunal Assemblage Associated with the Hominins (Australopithecus sediba) from the Early Pleistocene Cave Deposits of Malapa, South Africa
Source: PLoS One. 2015 Jun 10;10(6):e0126904. doi: 10.1371/journal.pone.0126904 (PMC4465193; doi:10.1371/journal.pone.0126904)
Supplement: S4 Table — (DOCX) [file pone.0126904.s013.docx]

**Table S4.**

| Specimen number | TAXONOMY | BONE | PORTION | SIDE |
| --- | --- | --- | --- | --- |
| UW88-802 | Felid (cf.*Dinofelis*) | radius | distal | right |
| UW88-803 | Felid (cf.*Dinofelis*) | radius | distal | left |
| UW88-643 | Lagomorph (*Lepus sp.*) | femur | complete | right |
| no number | Lagomorph (*Lepus* sp.) | femur | near complete | left |
| UW88-673 | Lagomorph (*Lepus* sp.) | ilium | complete | left |
| UW88-769 | Lagomorph (*Lepus* sp.) | pelvis | complete | right |
| no number | Lagomorph (*Lepus* sp.) | mandible with teeth | near complete | right |
| no number | Lagomorph (*Lepus* sp.) | mandible with teeth | near complete | left |
| no number | *Genetta* sp. | humerus | complete | right |
| no number | *Genetta* sp. | humerus | complete | left |
| UW88-748 | Bovid class II | femur | complete | right |
| UW88-1181 | Bovid class II | femur | near complete | left |
| UW88-1184 | Bovid class III | humerus | complete | right |
| UW88-1236 | Bovid class III | humerus | proximal | left |
| UW88-507 | Bovid class III | humerus | proximal | right |
| no number | Bovid class III | humerus | proximal | left |
| UW88-1213 | Bovid class III | radius | proximal | right |
| UW88-714 | Bovid class III | radius | proximal | left |
| UW88-1223 | Bovid class II | tibia | complete | right |
| no number | Bovid class II | tibia | complete | left |
| UW88-1247 | Bovid class III | metacarpal | complete | right |
| UW88-535 | Bovid class III | metacarpal | complete | left |
| UW88-1266 | Bovid class II | scapula blade | near complete | right |
| UW88-1234 | Bovid class II | scapula blade | near complete | left |
| UW88-518 and 519 | Bovid class III (*Tragelaphus* sp.) | mandible with teeth | near complete | right |
| UW88-929 | Bovid class III (*Tragelaphus* sp.) | mandible with teeth | near complete | left |
