# Supplementary material for: Taphonomic Analysis of the Faunal Assemblage Associated with the Hominins (Australopithecus sediba) from the Early Pleistocene Cave Deposits of Malapa, South Africa
Source: PLoS One. 2015 Jun 10;10(6):e0126904. doi: 10.1371/journal.pone.0126904 (PMC4465193; doi:10.1371/journal.pone.0126904)
Supplement: S5 Table — (DOCX) [file pone.0126904.s014.docx]

**Table S5.**

| Degree | Decalcified sediment | Calcified sediment |
| --- | --- | --- |
| Absent | 7 | 46 |
| Slight | 82 | 171 |
| slight to moderate | 17 | 17 |
| Moderate | 126 | 48 |
| moderate to heavy | 79 | 2 |
| Heavy | 29 | 4 |
