# Supplementary material for: Taphonomic Analysis of the Faunal Assemblage Associated with the Hominins (Australopithecus sediba) from the Early Pleistocene Cave Deposits of Malapa, South Africa
Source: PLoS One. 2015 Jun 10;10(6):e0126904. doi: 10.1371/journal.pone.0126904 (PMC4465193; doi:10.1371/journal.pone.0126904)
Supplement: S6 Table — (DOCX) [file pone.0126904.s015.docx]

**Table S6**.

| Stage | No. of specimens | % |
| --- | --- | --- |
| **1** | 243 | 34.4 |
| **2** | 170 | 24.0 |
| **3** | 127 | 18.0 |
| **4** | 129 | 18.2 |
| **5** | 38 | 5.4 |
